# Malaria vector diversity, transmission and insecticide resistance in Island communities along the Volta lake in Southern Ghana

**DOI:** 10.1186/s12879-025-11283-w

**Published:** 2025-07-09

**Authors:** Abena Ahema Ebuako, Christopher Mfum Owusu-Asenso, Anisa Abdulai, Abdul Rahim Mohammed Sabtiu, Isaac Kwame Sraku, Yaw Akuamoah-Boateng, Cornelia Appiah-Kwarteng, Akua Obeng Forson, Patrick Ferdinand Ayeh-Kumi, Yaw Asare Afrane

**Affiliations:** 1 Centre for Vector-Borne Disease Research, Department of Medical Microbiology, University of Ghana Medical School, Korle-Bu, Accra, Ghana; 2 Department of Medical Laboratory Science, School of Biomedical and Allied Health Sciences, University of Ghana, Korle-Bu, Accra, Ghana; 3 School of Veterinary Medicine, University of Ghana, Legon, Accra, Ghana

**Keywords:** Island communities, *Anopheles gambiae s.l*., Sporozoite rates, Entomological inoculation rate, Insecticide resistance

## Abstract

**Introduction:**

Island communities along the Volta Lake in southern Ghana present unique challenges for malaria control, characterized by high transmission rates, limited vector control measures and geographically isolated. This study assessed the malaria vector diversity, seasonal abundance, transmission and insecticide resistance status of malaria vectors in these communities to inform effective control strategies.

**Methods:**

Mosquitoes were collected from three Island communities (Tuanikope, Allorkpem and Pediatorkope) using human landing catches, light traps and prokopack aspirators during the dry and rainy seasons. Morphological and molecular techniques were used to identify mosquito species, determine blood meal sources and detect insecticide resistance mutations. Sporozoite infections and entomological inoculation rates (EIRs) were also quantified.

**Results:**

A total of 25,092 mosquitoes from four genera were collected (Culicine = 88.14%, Anopheline = 8.94% and *Mansonia* = 2.92%). The Anophelines predominantly comprised *Anopheles gambiae s.l.* (1,911/2,243, 85.20%) followed by *An*. *rufipes* (249/2,243, 11.10%) and *An. pharoensis* (83/2,243, 3.70%). Indoor biting and resting densities were high across sites and seasons, with sporozoite-positive mosquitoes more frequently found indoors. Blood meal analysis revealed a strong anthropophilic feeding pattern (HBI = 80%). Annual EIRs ranged from 37.40 (ib/m/y) to 100.08 (ib/m/y). Low frequencies of insecticide resistance mutations (*Vgsc-1014 F*, *Vgsc-1014 S*, *Ace-1* and *Vgsc-1575Y*) were observed.

**Conclusion:**

The study findings indicate high indoor biting and resting densities of *Anopheles* mosquitoes. High sporozoite rate along with low resistance mutation frequencies observed, emphasize the urgent need for continuous resistance monitoring and the implementation of targeted vector control strategies in these hard-to-reach Island communities.

**Supplementary Information:**

The online version contains supplementary material available at 10.1186/s12879-025-11283-w.

## Introduction

In Ghana, the *Anopheles gambiae sensu lato* and *Anopheles funestus* are the predominant malaria vector species [1–3]. The distribution of these vectors varies with changes in climate and ecological conditions, coupled with other factors such as land usage patterns and vector-host interactions [2, 4]. Island communities are characterized by a unique microclimate which may influence malaria transmission [5, 6]. Despite their geographical isolation from the mainland, these populations remain highly vulnerable to persistent malaria transmission due to a lack of vector control interventions, limited access to healthcare and the ecological suitability for mosquito breeding and proliferation [7, 8]. Malaria vectors in Island communities may undergo specific adaptations to the local ecosystem, such as changes in behaviour, physiology or genetics, reflecting the vectors’ vectorial capacity and ability to thrive in Island conditions [9].

Whilst several studies have reported on the malaria vector bionomics and insecticide resistance status across the mainland of Ghana within various ecological zones [3, 10–12], there is a paucity of data on the bionomics and insecticide resistance status of the malaria vectors in Island communities in Ghana. These Island communities face challenges distinct from those on the mainland, including the absence of electricity, which compels residents to spend extended hours outdoors during the evening, may increase human-vector contact and potentially influence malaria transmission dynamics and mosquito biting behaviour. The lack of structured vector control interventions [13, 14], such as long-lasting insecticide-treated nets (LLINs) and indoor residual spraying (IRS), further complicates the situation, as reduced insecticide pressure may alter mosquito resting and feeding behaviours. Moreover, the geographical isolation of these communities presents significant logistical barriers; access is only possible via river transport using small wooden boats, often under unpredictable weather and water conditions. This hampers routine vector surveillance, entomological monitoring, and the implementation of preventive and control interventions in these hard-to-reach communities [15] These limitations may translate to a high rate of malaria transmission due to the increased density of the disease vectors.

Hence, it is important to generate baseline data on vector bionomics and insecticide resistance specifically from these Island communities, which remain excluded from routine surveillance. These communities could serve as persistent reservoirs of infection and pose a risk of reintroduction even if malaria is successfully eliminated from the mainland. Up-to-date data from these areas are essential to guide the National Malaria Elimination Program in adapting and refining vector control strategies. This study assessed the malaria vector diversity, seasonal abundance, transmission and insecticide resistance status of malaria vectors in Island communities on the Volta Lake in Southern Ghana.

## Methodology

### Study sites

**Fig. 1:**
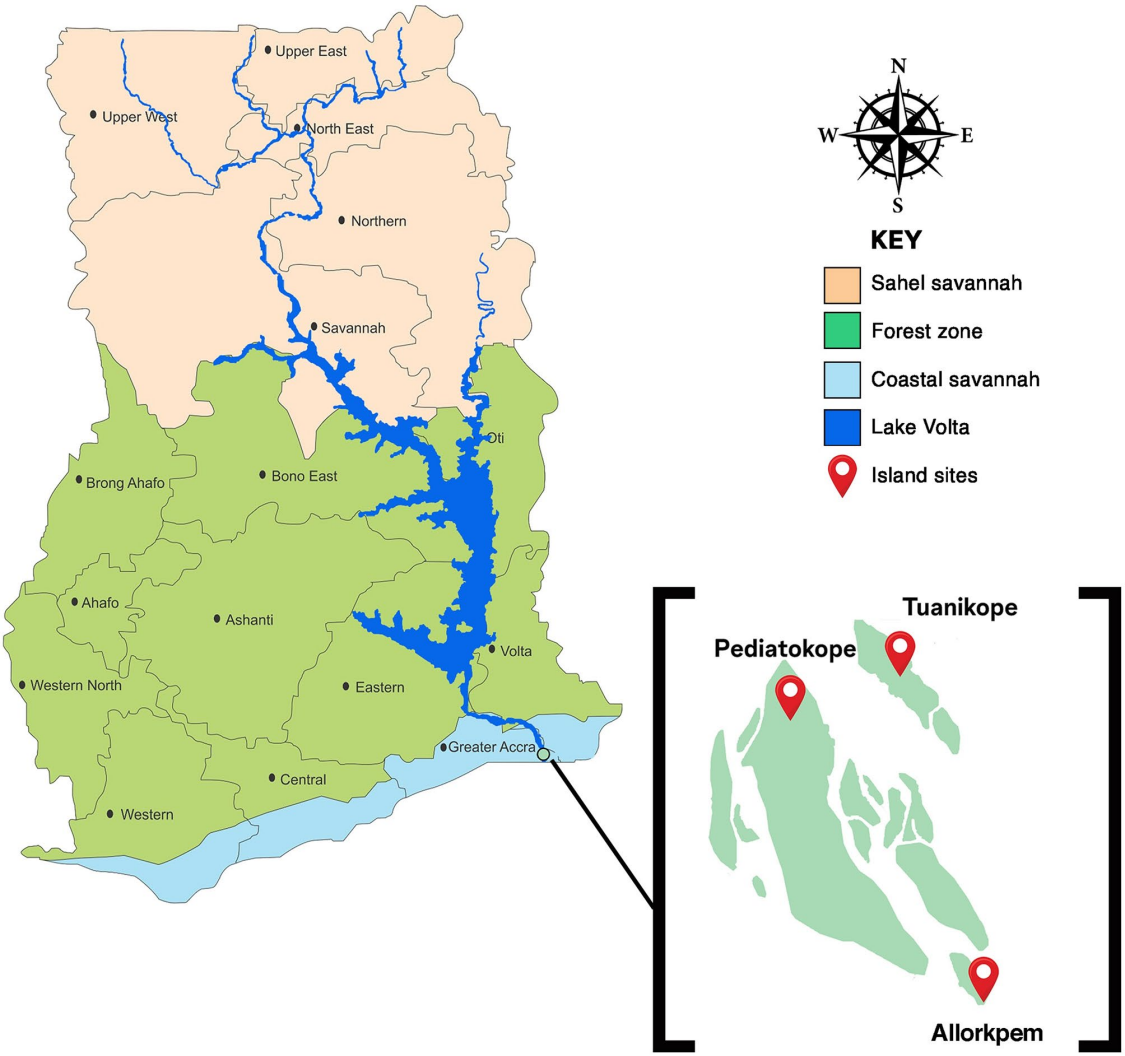
Map of Ghana showing study sites

The study was conducted in three Island communities; Tuanikope (W, 05.50.029 N, 000.38.778 E, 3), Pediatorkope (W, 05.48.844 N, 000.37.568 E, 6), and Allorkpem (W, 05.47.916 N, 000.39.141 E, 5), located on Lake Volta in the Ada East District of the Greater Accra Region in southern Ghana (Fig. 1). Each Island (Island community) is a standalone village community with permanent human settlements and distinct socio-environmental characteristics. Each village community comprises approximately 10,000–12,000 residents per village community [16]. The inhabitants of these three (3) sites are mainly engaged in fishing. These hard-to-reach Island communities are relatively isolated and accessible primarily by boat or canoe, with limited healthcare delivery.

Unlike the mainland settlements where the National Malaria Elimination Program (NMEP) regularly distributes long-lasting insecticidal nets (LLINs) and conducts indoor residual spraying (IRS), these Island populations are often excluded from such national campaigns probably due to the fact that they are hard-to-reach areas. Moreover, these communities have poor housing conditions, such as structures built from mud and thatch, offering minimal physical barriers to mosquito entry. Open eaves, lack of screened windows, and absence of ceilings may facilitate indoor mosquito resting and biting. The presence of stagnant water pools in abandoned fishing canoes, backyard gardening and poor drainage could provide ideal breeding habitats for *Anopheles* mosquitoes throughout the year.

In an earlier study, it was realized that these Island communities have not received vector control intervention for over eight years [13, 14]. Malaria transmission occurs all year round with seasonal peaks during the rainy season [13]. The communities lack electricity causing the inhabitants to spend longer hours outdoors, which could facilitate exposure to vectors.

These three Island communities lie in the coastal savannah in southern Ghana. It is characterized by a tropical savannah climate with temperatures ranging from 23 to 34 °C and an average annual rainfall of 787 mm, following a bimodal pattern with rainy seasons from April–June and October–November. The dry season lasts from December to March.

### Adult *Anopheles* mosquito collections using HLC, light traps and prokopack

At each sampling site, sixteen (HLC and light traps) and twenty (prokopack) houses were randomly selected and sampled by resting and biting locations (indoor and outdoor) during the dry (25th February– 8th March, 2023) and rainy (28th July– 5th August, 2023) seasons. At each site, HLC, light traps and prokopack collections were conducted in different houses. Houses previously sampled were excluded from subsequent sampling days to prevent repeated measurements and minimize sampling bias. *Anopheles* mosquitoes were collected for four nights in the dry and rainy season (four houses per day) per each Island community. Indoor and outdoor host-seeking mosquitoes were collected using the Human Landing Catch (HLC) technique [17] and light traps (John W. Hock Company, Gainesville, Florida, USA), whereas resting mosquitoes were collected using the prokopack aspirators (John W. Hock, Gainesville, FL). Each study site (Island community) was divided into 4 sections. Each section was sampled each day to ensure a fair representation of the mosquito population at each study site.

Individuals aged 18 years and above who volunteered to collect mosquitoes using the HLC method were trained to expose their lower limbs as bait to attract mosquitoes. Each night, the volunteers sat in the dark from 18:00 h– 6:00 h with their lower limbs exposed. With the aid of a flashlight, the trained volunteers located and collected host-seeking mosquitoes with a collection tube as the mosquitoes landed on their skin in search of a blood meal. To minimize collector bias and ensure quality control, two volunteers were positioned per house, one indoors and one outdoors, and rotated between locations every hour. This rotation helped balance mosquito attractiveness differences and maintain consistent sampling effort across collection sites. Additionally, volunteers took a 15-minute rest break at the end of each hour to reduce fatigue. All adult volunteers participating in the HLC study provided written informed consent before training and mosquito sampling. Volunteer recruitment was done from 21/02/2023 to 23/02/2023. Individuals who consented to partake in this study were given prophylaxis before the HLC collections. Independent staff supervised and regularly walked between different groups throughout the night, for quality control of collectors placed inside and outside selected houses [18].

The collection of mosquitoes using light traps took place in houses where the Human Landing Catch (HLC) method was not being used. Four houses were randomly selected for four nights and light traps were placed at a height of one meter from the ground of each house and baited with a mixture of sugar, yeast, and water to produce carbon dioxide to attract mosquitoes [19]. They were set up both inside and outside households at 18:00 h and retrieved at 06:00 h the following morning.

Each morning, the prokopack aspirator was used to hoover the ceiling, under tables, walls and all possible resting surfaces of mosquitoes in randomly selected houses, not previously sampled to collect any resting mosquitoes. Five houses were randomly sampled for four days between 5:30 h to 7:00 h during both seasons. Households were advised the night before sampling to refrain from opening their doors and windows upon waking the next morning to prevent resting mosquitoes from exiting their rooms.

### Morphological identification of *Anopheles* mosquitoes

All adult mosquitoes collected were knocked out by exposure to chloroform. Samples were sorted into the different genera and sex based on the identification keys by [20]. Female *Anopheles* mosquitoes were additionally categorized based on their gonotrophic status as unfed, blood-fed, half-gravid or gravid. All samples were individually packed on silica gel in Eppendorf tubes and labeled with the study site and date of collection [21]. Resting blood-fed *Anopheles* mosquitoes were placed separately in 1.5 ml Eppendorf tubes with 100% ethanol and then labeled. All samples were transported to the insectary of the Department of Medical Microbiology, University of Ghana Medical School in Korle Bu, Accra.

### Molecular identification of *An. gambiae s.l.*

A sub-sample from the total *An. gambiae s.l.* collected over the study period was selected according to study site, season and location (indoor or outdoor) and used to discriminate the species. Genomic DNA was extracted from individual mosquito legs and used as a template for species-specific PCR assays following the protocol of Scott et al. [22]. Amplified products were resolved on 2% agarose gels stained with ethidium bromide and visualized under UV light. Molecular forms of *A. gambiae s.s*. were further identified using primers targeting *A. coluzzii* and *A. gambiae s.s*. (Additional file 1).

### Detection of sporozoites

Genomic DNA extracted from the head and thoraces of sub-selected mosquitoes used for speciation were used to detect the presence of *Plasmodium* sporozoites by PCR as described by Echeverry et al. [23]. The primers, COX-IF and COX-IR were used to amplify a polymorphic fragment of the COX-(I) gene of the mosquito using a recombinant DNA polymerase (Additional file 1). To confirm the amplification of the 540 bp PCR product (indicating the presence of the *Plasmodium*), five microliters of the PCR product was electrophoresed on a 2% agarose gel and viewed under the UV Trans illuminator.

### Blood meal analysis in mosquitoes

To determine the vertebrate host origin of blood meals, genomic DNA was extracted from the abdomen of blood-fed Anopheles mosquitoes using the ZR DNA MicroPrep kit (Zymo Research), following the manufacturer’s protocol. Host DNA was identified by PCR amplification of the vertebrate mitochondrial cytochrome b gene using a set of five host-specific forward primers (human, cow, goat, pig, dog) and one universal reverse primer, as described by Kent and Norris [24]. Each PCR reaction included species-specific positive controls and a no-template negative control. PCR products were separated on a 2% agarose gel stained with ethidium bromide and visualized under UV light. The vertebrate source of the blood meal was determined based on the expected amplicon sizes: 334 bp (Human), 561 bp (Cow), 132 bp (Goat), 453 bp (Pig), and 680 bp (Dog). All primer sequences and amplification conditions are detailed in Additional file 1.

### Determination of insecticide-resistant mutations in *An. gambiae s.l.*

Genomic DNA was extracted from the heads and thoraces of adult *Anopheles* mosquitoes using the ZR DNA MicroPrep kit (Zymo Research) according to the manufacturer’s instructions. TaqMan SNP genotyping assays were performed to detect four key insecticide resistance mutations: *Vgsc-1014 F*, *Vgsc-1014 S*, *Vgsc-1575Y*, and *Ace-1 G119S*, following the protocol described by Bass et al. [25]. Each reaction was run in a 10 µL volume containing 5 µL of TaqMan Genotyping Master Mix (Applied Biosystems), 0.5 µL of each primer-probe mix, 2 µL of nuclease-free water, and 2 µL of genomic DNA. Genotyping was performed on a StepOnePlus™ Real-Time PCR System (Applied Biosystems), and allele discrimination was determined using allelic discrimination plots generated by the StepOne™ software v2.3. Primer and probe sequences, along with thermal cycling conditions, are provided in Additional file 1.

### Data management and analysis

Descriptive analysis was done to visualize insecticide resistant mutation frequencies, and mosquito species composition from the selected sites using graphs and tables. Chi-square analysis was used to determine if the data on two categories were associated. The number of female mosquitoes collected using the prokopack aspirator was used to calculate the resting density of Anopheline mosquitoes per site. The ratio of blood-fed mosquito samples that had drawn blood from people to the total number of mosquitoes screened for blood meal sources yielded the Human Blood Index (HBI). The sporozoite infection rate (SIR) was calculated as the total number of sporozoite-positive mosquitoes divided by the total number of mosquitoes tested. The total number of mosquitoes collected by HLC divided by the total number of collectors per total number of nights collected was used to compute the Human Biting Rate (HBR). The product of the sporozoite rate and the human biting rate was used to compute the Entomological Inoculation Rate (EIR). Allele frequencies of resistance gene markers in the vector populations at each site were calculated using Hardy-Weinberg equilibrium (HWE). All statistical analyses were done in and STATA/IC 14.1.

## Results

### Abundance and seasonal distribution of malaria vectors

Overall, 25,079 mosquitoes were collected belonging to four different genera during the sampling period. Mosquitoes collected in the dry season 17,638 (70.33%) were over twice as many as mosquitoes collected in the rainy season 7,441 (29.67%). Out of the total mosquitoes sampled, there were a total of 22,116 (88.19%) Culicines, 2,230 (8.89%) Anophelines and 733 (2.92%) *Mansonia*. The majority of Anophelines sampled were *Anopheles gambiae s.l.* 1,911 (85.70%) followed by *An. rufipes* 236 (10.58%) and *An*. *pharoensis* 83 (3.72%) (Table 1). Overall HLC [1,814 (94.92%)], was the most effective trapping method for sampling *An. gambiae s.l*., followed by light trap [73 (3.82%)], and prokopack [24 (1.26%)].

**Table 1:** Abundance and spatiotemporal distribution of mosquito genera and *Anopheles* species

Mosquitoes	Dry Season				Rainy Season			
	Allorkpem	Pediatorkope	Tuanikope	Total	Allorkpem	Pediatorkope	Tuanikope	Total
*An. gambiae s.l.*	706	252	592	1,550	155	76	130	361
*An. pharoensis*	2	1	46	49	8	8	18	34
*An. rufipes*	80	84	30	194	28	7	7	42
Culicine	5,792	5,350	4,605	15,747	2,691	1,173	2,505	6,369
*Mansonia*	84	3	11	98	34	166	435	635
Total	6,694	5,703	5,284	17,651	2,916	1,430	3,095	7,441

The overall abundance of Anopheline mosquitoes was significantly higher during the dry season 1,793 (80.40%) compared to the rainy season 437 (19.60%) (χ^2^ = 25.9274, df = 2, *P* < 0.001). *Anopheles gambiae s.l.* was the dominant species in both seasons, representing dry [*n* = 1,550/1,793; 86.45%] and rainy [*n* = 361/437; 82.61%]. *Anopheles rufipes* and *Anopheles pharoensis* were less abundant, constituting dry [194 (10.82%) vs. 49 (2.73%)] and rainy [42 (9.61%) vs. 34 (7.78%)] seasons, respectively (Table 1).

### Indoor and outdoor biting activity of *Anopheles* mosquitoes

A total of 23,984 mosquitoes [Anophelines. = 2,063 (8.60%), culicines = 21,194 (88.37%), *Mansonia* = 727 (3.03%)] were collected by the HLC method during the sampling period. Out of the total 2,076 Anophelines sampled, *An. gambiae s.l*. 1,814 (87.93%) were the most abundant, followed by *An. rufipes* 168 (8.14%) and then *An. pharoensis* 81 (3.93%). Overall, *Anopheles gambiae s.l*. demonstrated strong endophagic behaviour [indoor = 1,076 (59.32%) vs. outdoor = 738 (40.68%)]. However, *An. rufipes* [indoor = 102 (43.22%) vs. outdoor = 134 (56.78%)] and *An. pharoensis* [indoor = 38 (46.91%) vs. outdoor = 43 (53.09%)]. preferred feeding outdoors (exophagy) (Table 2).

**Table 2:** Biting location of *Anopheles *mosquitoes

Biting Location	Dry Season				Rainy Season			
	Allorkpem	Pediatorkope	Tuanikope	Total	Allorkpem	Pediatorkope	Tuanikope	Total
*An. gambiae s.l.*								
Indoor	467	174	296	937	97	56	66	219
Outdoor	239	78	296	613	58	20	64	142
Total	706	252	592	1,550	155	76	130	361
*An. pharoensis*								
Indoor	1	0	21	22	5	2	9	16
Outdoor	1	1	25	27	3	6	9	18
Total	2	1	46	49	8	8	18	34
*An. rufipes*								
Indoor	46	23	18	87	7	3	5	15
Outdoor	34	61	12	107	21	4	2	27
Total	80	84	30	194	28	7	7	42

### Human biting rate of *Anopheles* mosquitoes

The seasonal indoor human biting rate (HBR) of *An. gambiae s.l*. was higher than outdoor HBR in Allorkpem [dry (in = 13.78 vs. out = 7.41 mosquitoes/person/night) (m/p/n); rainy (in = 3.88 vs. out = 2.33 m/p/n)] (z = 0.166, *P* = 0.868). and Pediatorkope [dry (in = 4.97 vs. out = 2.38 m/p/n); rainy (in = 2.21 vs. out = 0.78 m/p/n)] (z = −0.440, *P* = 0.660). In contrast to the other sites, HBR in Tuanikope was higher outdoors than indoors during the dry season (in = 9.16 vs. out = 8.34 m/p/n). Whilst in the rainy season, the indoor HBR of *An. gambiae s.l.* remained higher than outdoors (in = 2.63 vs. out = 2.42 m/p/n).

### Biting patterns of *Anopheles gambiae s.l.*

**Fig. 2:**
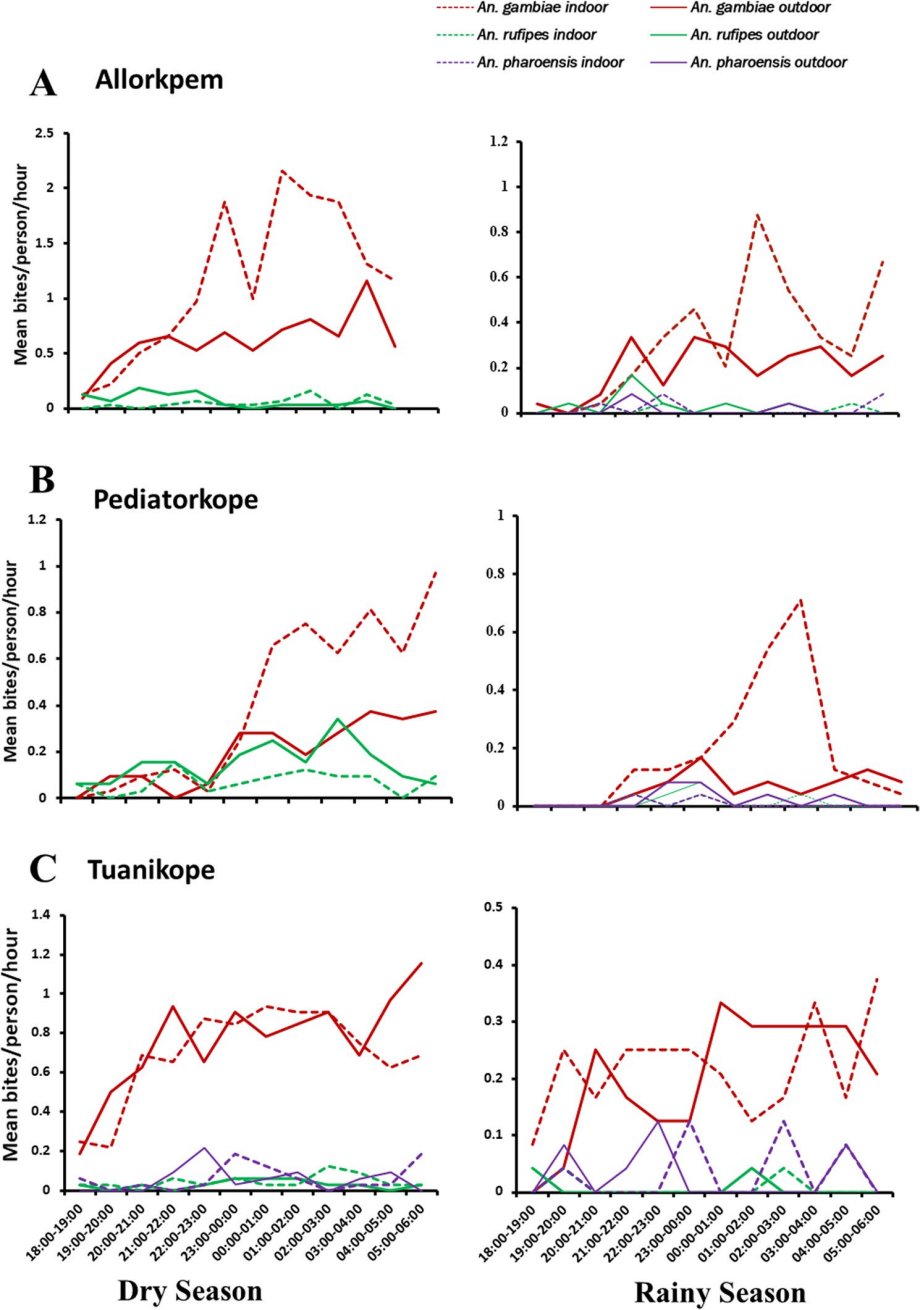
Hourly mean biting rate in (**A**): Allorkpem, (**B**): Pediatorkope and (**C**): Tuanikope per season

*Anopheles gambiae s.l.* had a preference for late evening/classical biting (LE: 22:00–4:00 h; 1134/1814; 66.2%) followed by early morning (EM: 4:00–6:00 h; 383/1814; 21.11%) and early evening (EE: 18:00–22:00 h; 297/1814; 16.37%) for both seasons. The pattern remained the same for indoors [LE (67.47%); EM (19.52%); EE (13.01%)] and outdoors biting [LE (55.28%), EM (23.44%); EE (21.27%)] across all study sites (Fig. 2).

High biting activity was also recorded beyond 06:00 h at all sites, suggesting extended host-seeking behaviour into daylight hours. Furthermore, biting patterns displayed multiple peaks throughout the night. This was observed across all three Island communities and in both seasons (Fig. 2).

### Resting densities of *Anopheles gambiae s.l.*

**Fig. 3:**
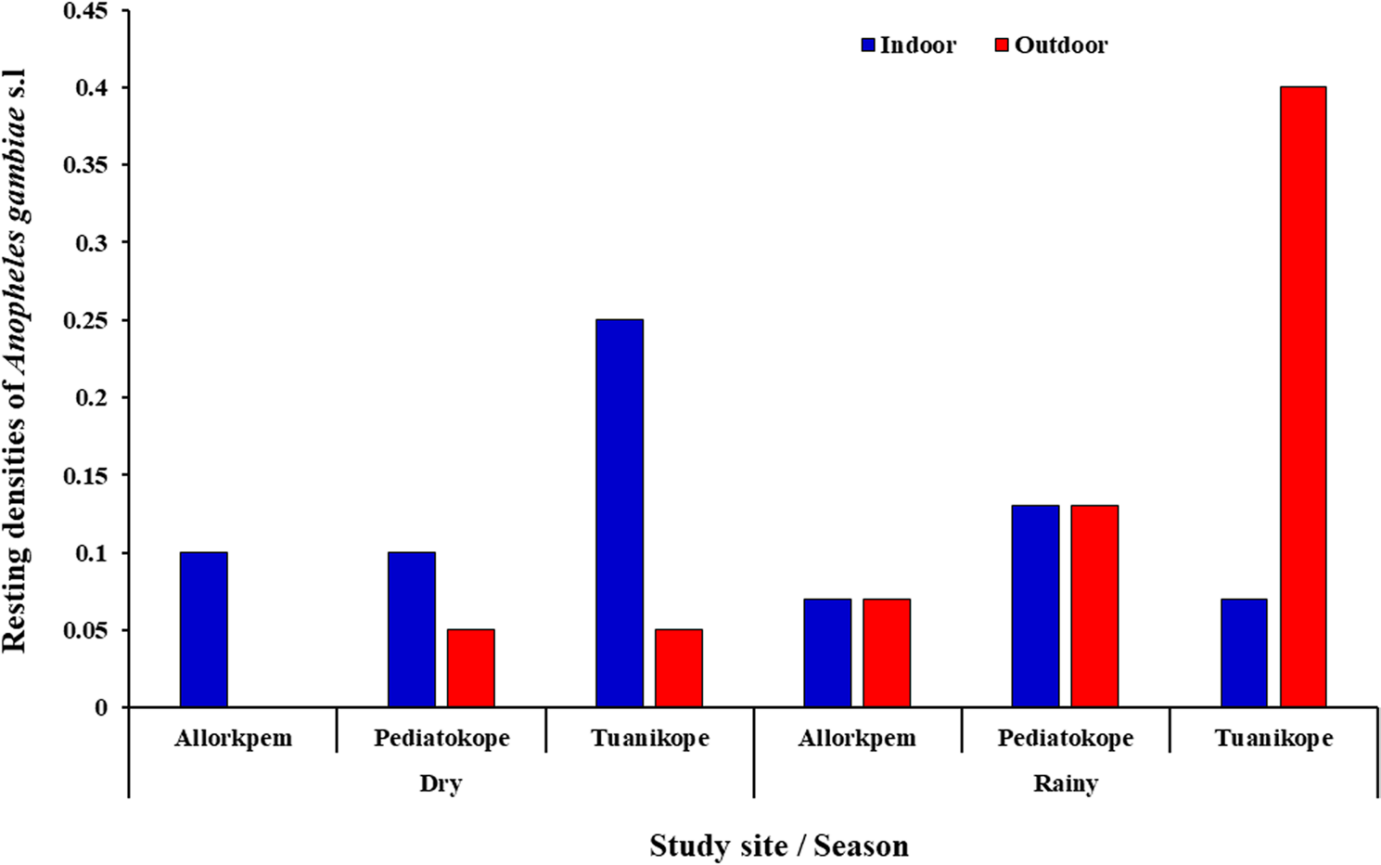
Resting densities of *An. gambiae s.l.* collected from Island communities

Out of the 1,911 *An*. *gambiae s.l.* collected; 24 resting mosquitoes were caught using the prokopack aspirator. Overall, majority of the resting mosquitoes were collected during the rainy season accounting for 52.17% (13/24) compared to 47.83% (11/24) collected during the dry season. Majority of the mosquitoes were sampled indoors (52.17%) whiles 47.83% were collected outdoors. During the dry season, the highest indoor resting density was recorded in Tuanikope (5/11; 0.25%). Similarly, the highest resting density during the rainy season was recorded outdoors in Tuanikope (6/13; 0.4%) (Fig. 3).

### Species discrimination of the *An. gambiae* complex

**Fig. 4:**
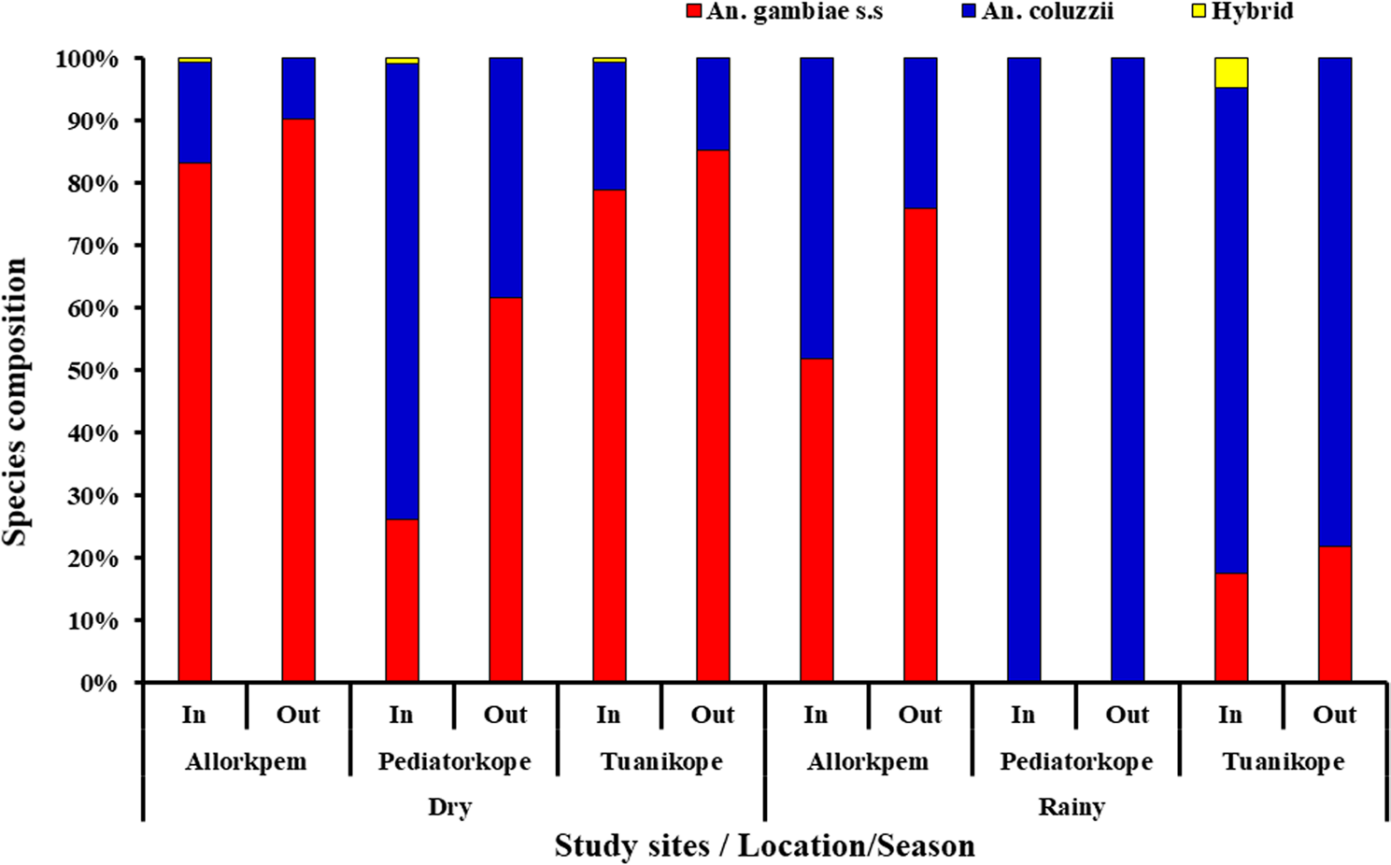
Species discrimination in the *Anopheles gambiae s.l.*

From the total *An*. *gambiae s.l.* collected across all sites, a subsample of 862 were randomly selected and used to distinguish between the sibling species. This resulted in 538 (62.88%) *An. gambiae s.s*., 316 (37.04%) *An. coluzzii* and 8 (0.94%) hybrids, (Fig. 4). The species composition differed significantly across seasons (χ^2^ = 124.3045.92, *df* = 2, *P* = 0.000), resting and biting location (indoor/outdoor) (χ^2^ = 39.8512, *df* = 2, *P* = 0.000), and study sites (χ^2^ = 149.4988, df = 4, *P* = 0.000). Overall, *Anopheles gambiae s.s*. dominated mosquito populations across Island communities, except in Pediatorkope (*An*. *gambiae s.s.* = 36/158; *An*. *coluzzii* = 122/158), where *An. coluzzii* were more abundant (χ^2^ = 283.27, df = 6, *P* = 0.000).

### Sporozoite infectivity rates in the sampled vectors

In addition, all 862 sub-samples that were genotyped to species were used to determine the presence of sporozoites. Thirty-one (31) samples [dry = 15/640; rainy = 16/222] (χ^2^ = 2.2485, *df* = 1, *P* > 0.05) were positive for *P. falciparum* CSP, giving an overall sporozoite rate (SR) of 0.030. Infected mosquitoes were identified from all three Island communities: Allorkpem: [SR = 0.02; *n* = 8/326], Pediatorkope: [SR = 0.04; *n* = 6/160] and Tuanikope: [SR = 0.05); *n* = 17/376], with no significant variation in sporozoite rate (χ^2^ = 0.1690, *df* = 1, *P* > 0.05). Though more CSP-positive *An*. *gambiae s.l*. mosquitoes were collected indoors [SR = 0.61; *n* = 19/31] than outdoors [0.39; *n* = 12/31], the difference was not significant (χ^2^ = 0.0937, *df* = 1, *P* > 0.05). Overall, majority of the infected mosquitoes were *Anopheles coluzzii* (*n* = 20/31) and were collected during the classical biting time (00:00 h– 3:00 h). No sporozoite infections were detected in other sibling species of the *An. gambiae* complex, though they are known transmitters of malaria.

### Entomological inoculation rates in the sampled vectors

Overall, Tuanikope had the highest estimated annual EIR of 100.08 (ib/m/y) followed by Allorkpem [65.94 (ib/m/y)] and Pediatorkope [37.40 (ib/m/y)]. In the dry season, an unprotected individual living in Allorkpem was most exposed to an infective bite from *Anopheles* mosquito with an EIR of 0.26 ib/m/n. Tuanikope 0.19 (ib/m/n) followed as the next highest risk area, with Pediatorkope 0.11 (ib/m/n) having the lowest exposure among the three locations. Conversely, in the rainy season, individuals in Tuanikope were more exposed to infective bites with an EIR of 0.28 ib/m/n followed by Pediatorkope 0.11ib/m/n and Allorkpem 0.07 ib/m/n. (Table 3).

**Table 3:** Entomological inoculation rate for Island communities

Study sites	Season	HBR(b/*p*/*n*)	SPR	EIR(ib/m/*n*)	EstEIR/y(ib/m/y)
Allorkpem	Dry	10.59	0.03	0.26	96.63
	Rainy	3.1	0.02	0.07	27.16
	TOTAL	7.38	0.02	0.18	65.94
Pediatorkope	Dry	3.67	0.03	0.09	33.49
	Rainy	1.48	0.08	0.11	40.52
	TOTAL	2.73	0.04	0.10	37.40
Tuanikope	Dry	8.75	0.02	0.19	70.26
	Rainy	2.52	0.11	0.28	101.18
	TOTAL	6.08	0.05	0.27	100.08

*HBR* man biting rate, *SPR* sporozoite rate, *EIR* entomological Inoculation rate, *ESTEIR/y* estimated entomological inoculation rate per year, *ib/m/n* infective bite per man per night, *b/p* number of mosquito bites per person, *ib/m/y* infective bites per man/year

### Blood meal analysis in sampled malaria vectors

A total of 15 blood-fed resting adult *An. gambiae s.l.* samples caught during the dry (Allorkpem *n* = 3, Pediatorkope *n* = 2 and Tuanikope *n* = 1) and rainy season (Allorkpem *n* = 0, Pediatorkope *n* = 1 and Tuanikope *n* = 8) were analyzed to determine their blood meal sources. Out of these, 9 (60%) fed on humans, 5 (33.3%) fed on goats and pigs and 1 (6.67%) neither fed on humans, goats or pigs. It was identified by PCR, that 80% of the HBI was detected from indoor collected *An. coluzzii*, indicating its primary role in malaria transmission in the study areas. No blood-fed mosquitoes were detected among other sibling species of the *An. gambiae* complex. The primary source of animal blood meals was identified as goats. A mixed blood meal source (human and goat) was identified from a single indoor mosquito. Additionally, mixed pig and goat bloodmeal were found in one indoor and one outdoor mosquito. Among the sites, a higher indoor HBI was recorded in Tuanikorpe (83.3%), followed by Allorkpem (33.3%)] and then Pediatorkope (0%) (Table 4).

**Table 4:** Blood meal sources of resting *An. coluzzii* per site

Site	Blood-meal origins	*An. coluzzii*	
		Indoor *n* (%)	Outdoor *n* (%)
Allorkpem	Number Tested	3	0
	Human	1 (33.3)	0
	Goat	2 (66.7)	0
	HBI	33.3	0
	ABI	66.7	0
Pediatorkope	Number Tested	2	1
	Human	0	0
	Goat	1 (50)	1 (100)
	Un-identified	1 (50)	0
	HBI	0	0
	ABI	100	100
Tuanikope	Number Tested	6	3
	Human	3 (50)	1 (33.3)
	Goat	1 (16.7)	1 (33.3)
	Human + Goat	1 (16.7)	0
	Human + Pig	1 (16.7)	1 (33.3)
	HBI	83.3	66.7
	ABI	50	66.7

*HBI* human blood index, *ABI* animal blood index, *HBI* human blood index, *ABI* animal blood index

### Insecticide resistant mutations in *Anopheles gambiae s.l.*

Low frequencies of *kdr* mutations assayed were observed in *Anopheles* mosquitoes from all sites; *vgsc-1014 F* (0.02–0.03), *vgsc*-*1014 S* (0.00–0.04) and *vgsc*-*1575Y* (0.06–0.35). Similarly, low frequencies of *Ace-1* mutation was observed in *Anopheles* mosquitoes across all sites (0.03–0.06) (Table 5).

**Table 5:** Frequency and distribution of resistance genes in *Anopheles *mosquitoes collected from Island communities along the Volta Lake in Southern Ghana

Study site	Location	*Vgsc-1014 F*		*Vgsc-1014 S*		*Vgsc-1575Y*		*Ace-1*	
		N	F	N	F	N	F	N	F
Allorkpem	Indoor	200	0.03	200	0.00	50	0.21	50	0.04
	Outdoor	127	0.03	127	0.04	50	0.35	50	0.03
	Total	327	0.03	327	0.01	100	0.28	100	0.04
Pediatokope	Indoor	118	0.00	118	0.00	50	0.07	50	0.06
	Outdoor	42	0.00	42	0.02	50	0.06	50	0.06
	Total	160	0.00	160	0.01	100	0.07	100	0.06
Tuanikope	Indoor	202	0.03	202	0.01	50	0.30	50	0.06
	Outdoor	175	0.00	175	0.00	50	0.12	50	0.03
	Total	377	0.02	377	0.01	100	0.21	100	0.05

*N* Sample size, *F* Allele frequency

## Discussion

Understanding the bionomics, spatiotemporal distribution and behavioural changes of malaria vectors in Island communities is essential for designing targeted vector control measures. This study investigated the biting and resting behaviour, rates of infection with *Plasmodium falciparum* sporozoites, blood meal sources and frequencies of insecticide resistance alleles of mosquitoes collected from Allorkpem, Pediatorkope and Tuanikope. Higher densities of *Anopheles* mosquitoes were collected during the dry season. A high sporozoite rate with a low prevalence of *kdr* mutations was observed in *Anopheles* mosquitoes.

*An. gambiae s.l.* was the predominant vector for malaria transmission in Island communities year-round. However, its abundance exhibited significant seasonal variation. The abundance of *Anopheles gambiae s.l.* was significantly higher during the dry season compared to the rainy season. While existing literature often correlates rainy seasons with increased mosquito populations due to expanded breeding sites [26, 27], the extreme rains, coupled with windy conditions observed on the Islands during the rainy season, may have flooded the breeding habitats and washed off the larvae [28], potentially leading to the lower adult abundance.

Malaria vectors have been efficient in transmitting malaria mainly due to their anthropophilic and endophilic behaviour. Therefore, knowledge on the biting behaviour of malaria vectors in local vector populations is important for deploying effective control tools and interrupting disease transmission. In this study, the abundance of *Anopheles* mosquitoes biting and resting indoors was higher than outdoors in both seasons. Studies on the mainland in Ghana observed a high abundance of *Anopheles* mosquitoes biting outdoors [18, 29, 30]. The high indoor biting and resting mosquitoes may be attributed to the absence of key vector control interventions such as LLINs and IRS. Long-Lasting Insecticide Nets and IRS are cornerstone strategies in malaria prevention, effectively reducing mosquito densities and malaria transmission by targeting endophilic (indoor-resting) and anthropophilic (human-biting) vectors [31–33]. Their absence likely creates an unprotected environment that facilitates the abundance of mosquitoes within households. Moreover, outdoor biting mosquitoes could maintain residual malaria transmission [18]. Hence, continuous monitoring of mosquito behaviour is crucial to developing effective malaria control interventions.

The biting behaviour of *Anopheles gambiae s.l.* observed across the Island communities demonstrated biting activity extending into the early hours of the morning, with continued activity even beyond 06:00 h. Such extended biting activity may pose significant challenges for LLINs use, which are primarily designed to offer protection during typical sleeping hours. This vector behaviour raises serious public health concerns, as this coincides with the dawn activities of the inhabitants, as residents wake up as early as 02:00–03:00 h to fish, fetch water and firewood, and tend to animals. This creates a window of high vulnerability for residual malaria transmission and may potentially hinder control efforts in the region [34]. Similar behaviour has been reported in other parts of sub-Saharan Africa, with a shift in biting times in response to selective pressure from LLINs and IRS interventions [35, 36]. However, in the studied Island communities, characterized by minimal insecticide pressure due to lack of LLIN and IRS coverage, such biting plasticity may reflect intrinsic behavioural traits or ecological adaptation rather than behavioural resistance.

*Anopheles coluzzii*, the predominant species in this study, demonstrated a strong tendency to bite indoors during the late hours of the night, consistent with its known endophagic behaviour reported in other studies [37, 38]. Another finding from this study was the presence of other secondary malaria vectors biting mainly outdoors: *An. rufipes* and *An. pharoensis*. These vectors have been implicated in other areas in transmitting malaria [39–41]. While their specific contribution to malaria transmission in Ghana is not fully known, their presence emphasizes the complex nature of malaria transmission in the study area. Hence, the need for a comprehensive vector control strategy. These species-specific biting patterns have critical implications for malaria transmission, particularly in communities lacking LLINs and IRS, where human exposure remains high throughout the night.

Sporozoite rates were observed to be relatively similar in both seasons across all study sites. Nonetheless, the higher proportion of sporozoite-positive *Anopheles coluzzii* collected indoors compared to outdoors is particularly concerning, given the absence of indoor vector control interventions such as LLINs and IRS. This finding suggests sustained malaria transmission throughout the year, irrespective of seasonal changes. Without these interventions, indoor environments remain unprotected, allowing mosquitoes to thrive, feed, and rest. This may lead to a higher likelihood of malaria transmission within households on these Island communities, implying a consistent level of malaria transmission throughout the year [12, 42].

The entomological inoculation rate (EIR) remains a relevant index for assessing malaria transmission intensity and endemicity [43, 44]. Among the study sites, Tuanikope recorded the highest EIR, attributable to its high human biting rate (HBR) and sporozoite rate prevalence. Comparatively, other studies conducted in rural and urban areas across Africa have reported annual EIRs ranging from 0 to 884 and 0 to 43 infectious bites per person (m/b/p) respectively [45, 46]. The findings of this study highlight high malaria transmission in Island communities, where residents lack adequate vector control measures and may experience relatively high annual EIRs (37.40 m/b/p to 100.08 m/b/p) across all study sites. These values significantly exceed the EIR of 21.9 m/b/p previously reported in Ghana’s coastal forest zone [47], although they remain below the EIR of 163 m/b/p reported on Bioko Island, Guinea Bissau [6]. These data necessitate the urgent need for enhanced malaria control interventions in these vulnerable Island populations.

Analysis of blood meals indicated a significant portion of malaria vectors displaying anthropophagic behaviour. The increased proportions of infected bloodfed *Anopheles coluzzii* resting indoors raise concerns for malaria elimination initiatives, given the effectiveness of these mosquitoes in transmitting malaria [35, 48, 49].

The observed low frequency of resistance mutations in outdoor and indoor biting mosquito populations could be attributed to the absence of vector control tools in these areas, which could limit mosquito exposure to insecticides. During the study it was realized that no vector control campaigns has been done in the area for close to a decade, probably due to the hard-to-reach nature of the study sites. Without interventions such as IRS or LLINs, vectors may not be subjected to lethal or sub-lethal doses of insecticides, including those from alternative sources like aerosol sprays or agricultural pesticides [50]. This lack of exposure, combined with other factors influencing insecticide resistance, may contribute to the observed low allelic frequencies of resistant mutations in this study. This observation suggests that the majority of the mosquito population in the study sites may be susceptible to insecticides used for vector control. A key limitation of this study was the absence of phenotypic resistance data, largely due to limited larval availability. Despite the short sampling period across the dry and rainy seasons, this exploratory study offers valuable preliminary insights into the species composition, biting behaviour, resistance status, and transmission potential of malaria vectors in these island communities. These findings provide critical baseline data to inform future policy decisions and guide the development of targeted vector control interventions.

## Conclusion

This study reveals sustained malaria transmission in hard-to-reach Island communities on Lake Volta, primarily driven by *Anopheles coluzzii*. High indoor biting and resting densities, extended outdoor and early morning host-seeking activity, and a high human blood index (HBI: 80%) were observed. Despite low resistance mutation frequencies, the absence of structured vector control interventions necessitates the urgent need for continuous resistance monitoring and the implementation of targeted vector control strategies in these communities.

## Supplementary Information

Additional file 1: A table showing details of primer sequences, band sizes and thermocycling conditions for molecular assays

## Data Availability

All the data supporting this study are included in the article.

## Abbreviations

DNA: Deoxyribonucleic acid
PCR: polymerase chain reaction
An.: Anopheles
CO2: Carbon dioxide
GPS: geographical position system
HBI: Human blood index
HLC: Human landing catch
PPK: prokopack
WHO: World Health Organization
