# Supplementary material for: Malaria vector diversity, transmission and insecticide resistance in Island communities along the Volta lake in Southern Ghana
Source: BMC Infect Dis. 2025 Jul 9;25:904. doi: 10.1186/s12879-025-11283-w (PMC12243191; doi:10.1186/s12879-025-11283-w)
Supplement: Supplementary file 1 — Additional file 1: A table showing details of primer sequences, band sizes and thermocycling conditions for molecular assays [file 12879_2025_11283_MOESM1_ESM.docx]

| **Aditional Table 1: Table showing details of primer sequences, band sizes and thermocycling conditions for molecular assays** | | | |
| --- | --- | --- | --- |
|  |  |  |  |
| **Primer** | **Primers Sequence (5’-3’)** | **Band size** | **Cycling Conditions** |
| ***Anopheles gambiae s.l*. Specie Discrimination** | | | |
| **universal** | GTGTGCCGCTTCCTCGATGT |  | 95°C/3min x 1 cycle, (95°C/30sec) (54°C/30sec, 72°C/1min) x 35 cycles, 72°C/5min x 1 cycle, 4°C hold. |
| ***Anopheles gambiae* s.l** | CTGGTTTGGTCGGCACGTTT | 390 |  |
| ***Anopheles arabiensis*** | AAGTGTCCTTCTCCATCCTA | 315 |  |
| ***Anopheles melas*** | GTGACCAACCCACTCCCTTGA | 464 |  |
| ***Anopheles gambiae M & S form*** |  |  |  |
| **F6.1a** | TCGCCTTAGACCTTGCGTTA | 500 (M)  260 (S) | 95°C/3min x 1 cycle, (95°C/30sec) (54°C/30sec, 72°C/1min) x 35 cycles, 72°C/5min x 1 cycle |
| **F6.1b** | CGCTTCAAGAATTCGAGATAC |  |  |
| **Sporozoite Detection in *Anopheles gambiae s.l.*** | | | |
| **Cox -I F** | AGAACGAACGCTTTT AACGCCTG | 540 bp | 94 °C for 5 minutes followed by 40 cycles of 94 °C for 1 min, 62 °C for 1 min and 72 °C for 90 s, and a final elongation step at 72 °C for 10 min. |
| **Cox -I R** | ACTTAATGGTGGATATAAAGTCCATCCWGT |  |  |
| **Bloodmeal Sources in *Anopheles gambiae s.l.*** | | | |
| **UNI Rev** | GGTTGTCCTCCAATTCATGTTA |  | 94℃ for 3 minutes  (94℃ for 30 seconds  62℃ for 30 seconds cycles, 72℃ for 30 seconds) x 35 cycles  72℃ for 5 minutes |
| **Goat 894F** | CCTAATCTTAGTACTTGTACCCTTCCTC | 132 |  |
| **Human 741F** | GGCTTACTTCTCTTCATTCTCTCCT | 334 |  |
| **Pig 573F** | CCTCGCAGCCGTACATCTC | 453 |  |
| **Dog 368 F** | GGAATTGTACTATTATTCGCAACCAT | 680 |  |
| **Cow 121 F** | CATCGGCACAAATTTAGTCG | 561 |  |
|  |  |  |  |
|  |  |  |  |
|  |  |  |  |
| ***Kdr* Mutation Detection in *Anopheles gambiae s.l.*** | | | |
| **AgD1** | ATAGATTCCCCGACCATG | SS (293 and 137)  RS (293, 195 and 137)  RR (2 ,93 and 195) | a 94°C/5min x 1 cycle, (94°C/1min, 48°C/2min, 72°C/2min) x 40 cycles, 72°C/10min x 1 cycle, 4°C hold |
| **AgD2** | AGACAAGGATGATGAACC |  |  |
| **AgD3** | AATTTGCATTACTTACGACA |  |  |
| **AgD4** | CTGTAGTGATAGGAAATTTA |  |  |
| **AgD5** | TTTGCATTACTTACGACTG |  |  |
| **Ace-1 Mutation Detection** | | | |
| **ace-1-F** | GGCCGTCATGCTGTGGAT | SS (540 and 139)  RS (540. 253 and 139)  RR (253, 150 and 139) | 95°C for 2 min; 11 cycles of 94°C for 20 s, 65°C for 40 s, and 72°C for 1.5 min; 24 cycles of 94°C for 20 s, 59°C for 30 s, and 72°C for 1.5 min; and 2 min at  72°C |
| **ace-1-R** | TGGCGGTGCCGGAGTAGA |  |  |
